# Supplementary material for: NTPDase1/CD39 Ectonucleotidase Is Necessary for Normal Arterial Diameter Adaptation to Flow
Source: Int J Mol Sci. 2023 Oct 10;24(20):15038. doi: 10.3390/ijms242015038 (PMC10606763; doi:10.3390/ijms242015038)
Supplement: Supplementary file 1 [file ijms-24-15038-s001.zip › ijms-2607050-supplementary.pdf]

Supplementary Figure S1

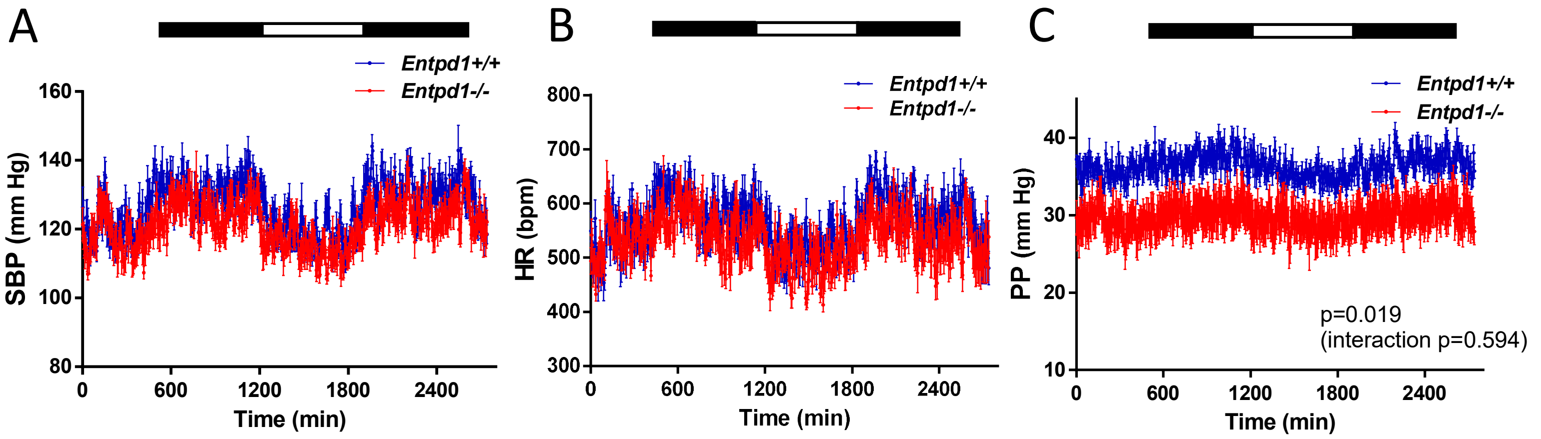

Supplementary Figure S1: *In vivo* hemodynamic parameters assessed by radiotelemetry in *Entpd1*$^{+/+}$ (blue) and *Entpd1*$^{-/-}$ (red) mice. Variations over periods (12h) of dark (black square) and light (white square). (A) Systolic blood pressure (SBP); (B) Heart rate (HR); (C) Pulse pressure (PP). Two-way ANOVA for repeated measurements was performed (*Entpd1*$^{-/-}$ versus *Entpd1*$^{+/+}$ mice) over 48h of measurements and significant p values are shown on graphs when significant.

Supplementary Figure S2

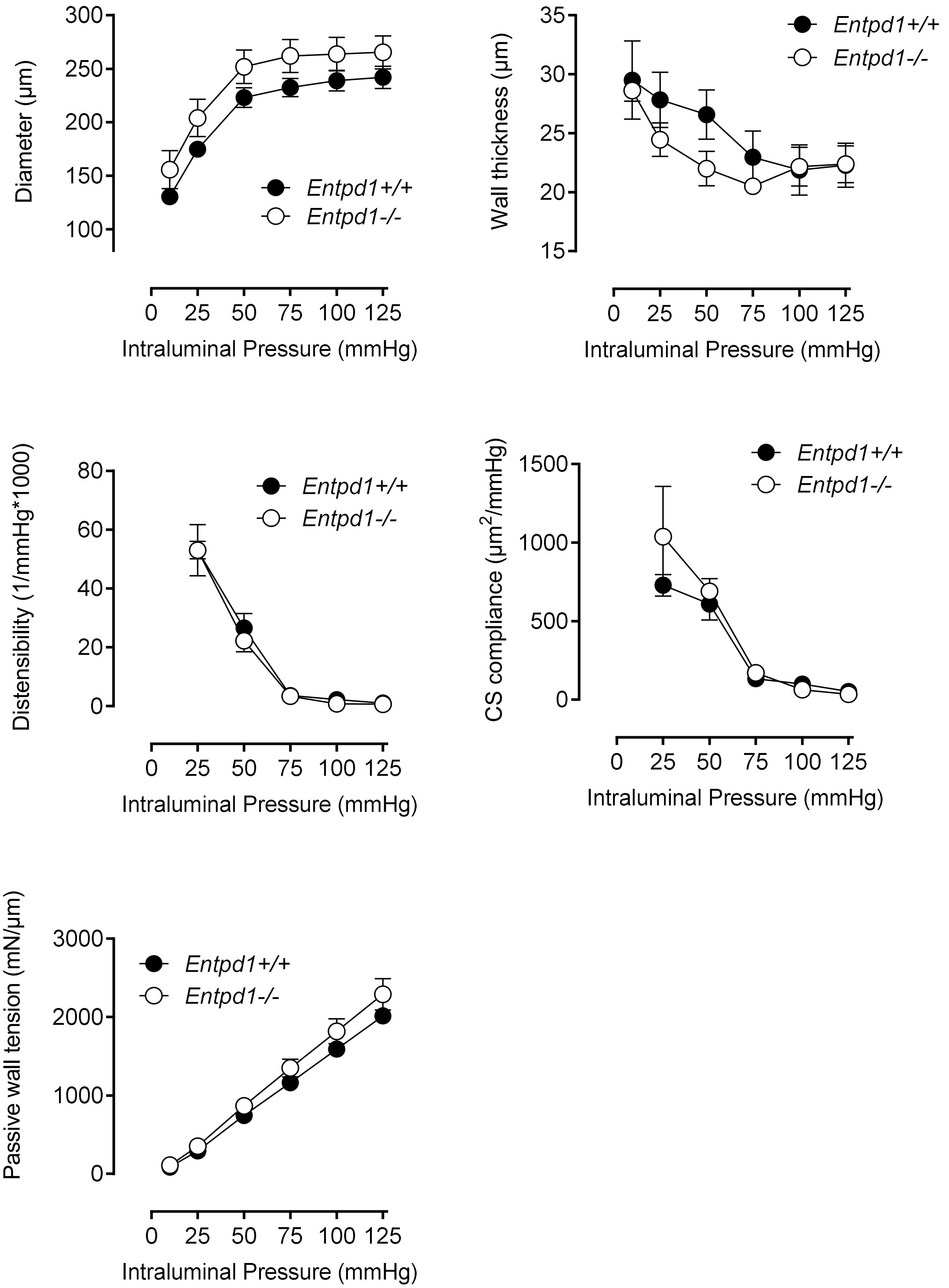

Supplementary Figure S2: Impact of Entpd1 deletion on mechanical properties assessed on isolated pressurized MRA. Passive changes in arterial diameter in response to the increase in internal pressure were measured on MRA superfused in PSS physiological solution without calcium with EGTA. Two-way ANOVA for repeated measurements was performed (*Entpd1*$^{-/-}$ versus *Entpd1*$^{+/+}$ mice, not significant).

Supplementary Figure S3

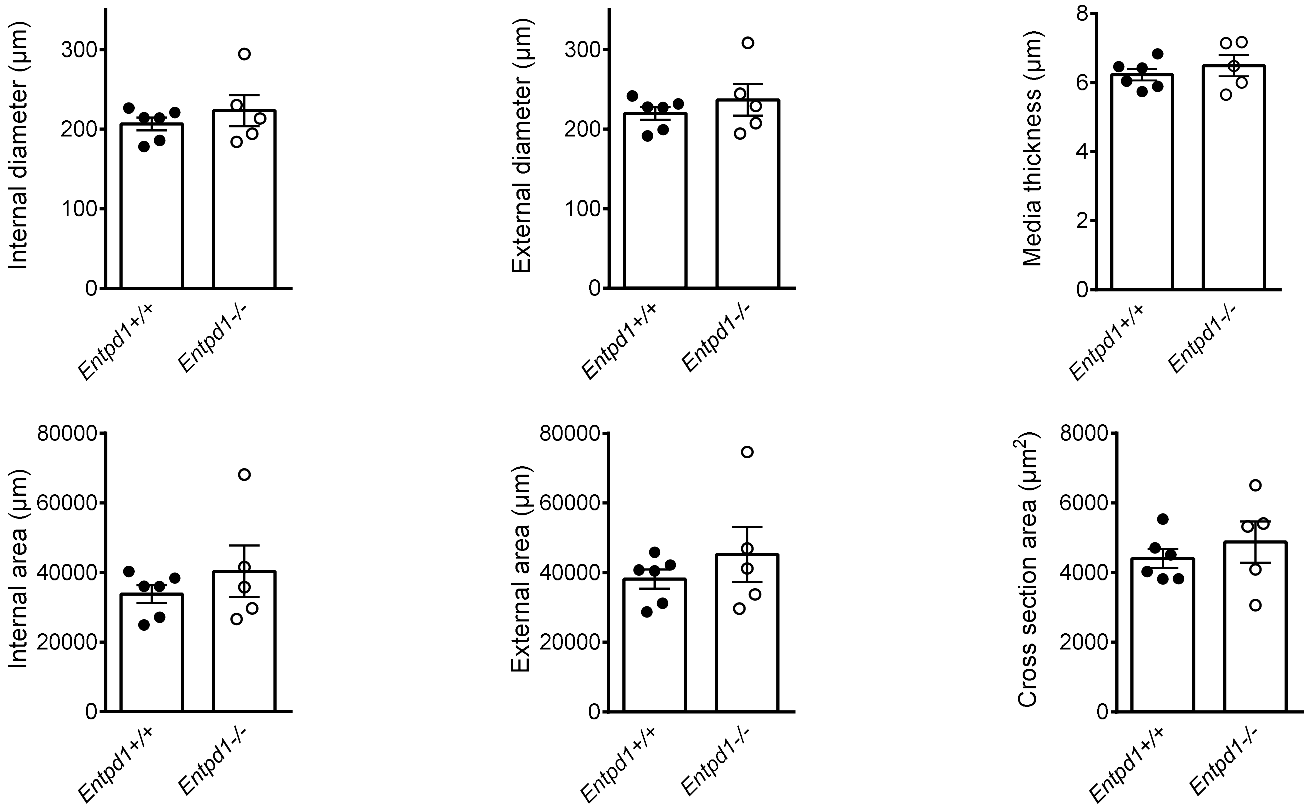

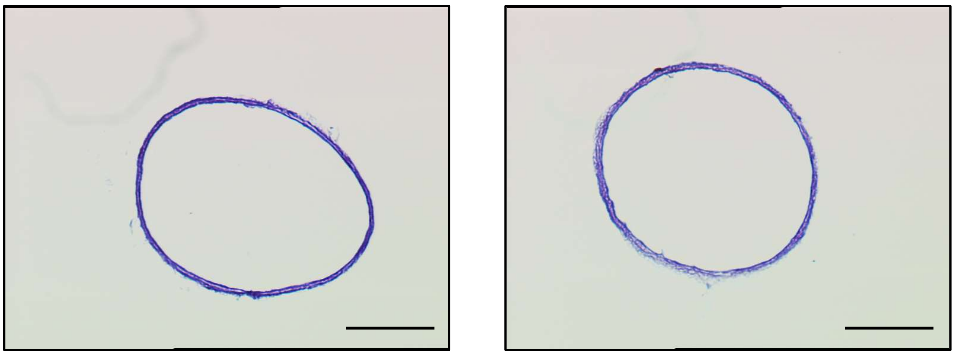

Entpd1+/+ *Entpd1-/-*

Supplementary Figure S3: Histomorphological analysis of pressurized mesenteric arteries. The MRA isolated from male *Entpd1-/-* and their wild-type littermates *Entpd1+/+* were cannulated and pressurized at 75 mmHg in physiologic solution without Ca^{2+} in order to obtain maximal passive diameter and the arteries were then fixed with PFA 4% before being embedded for histological staining with orcein. Scale bar 100 µm.

Supplementary Figure S4

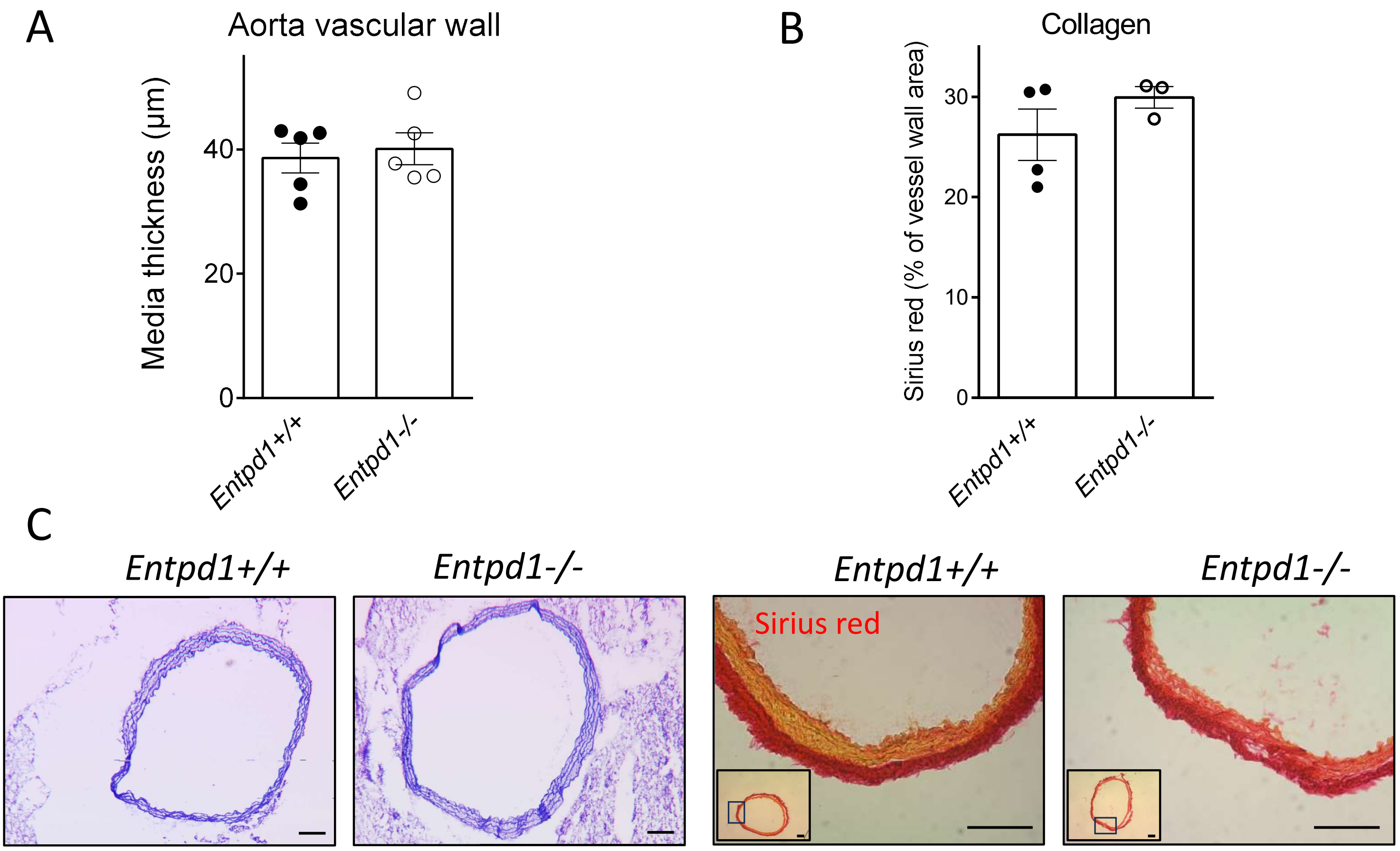

Supplementary Figure S4: Aorta vascular wall structure (A) and collagen content (B). Image J software was used to quantify media thickness (orcein staining) and collagen (Sirius red, expressed as % area of stained vessel wall). (C) Images illustrating histological analysis of thoracic aorta of *Entpd1-/-* and their control littermates *Entpd1+/+.* Different magnifications are shown for Sirius red staining. Scale bar 100 µm..

Supplementary Figure S5

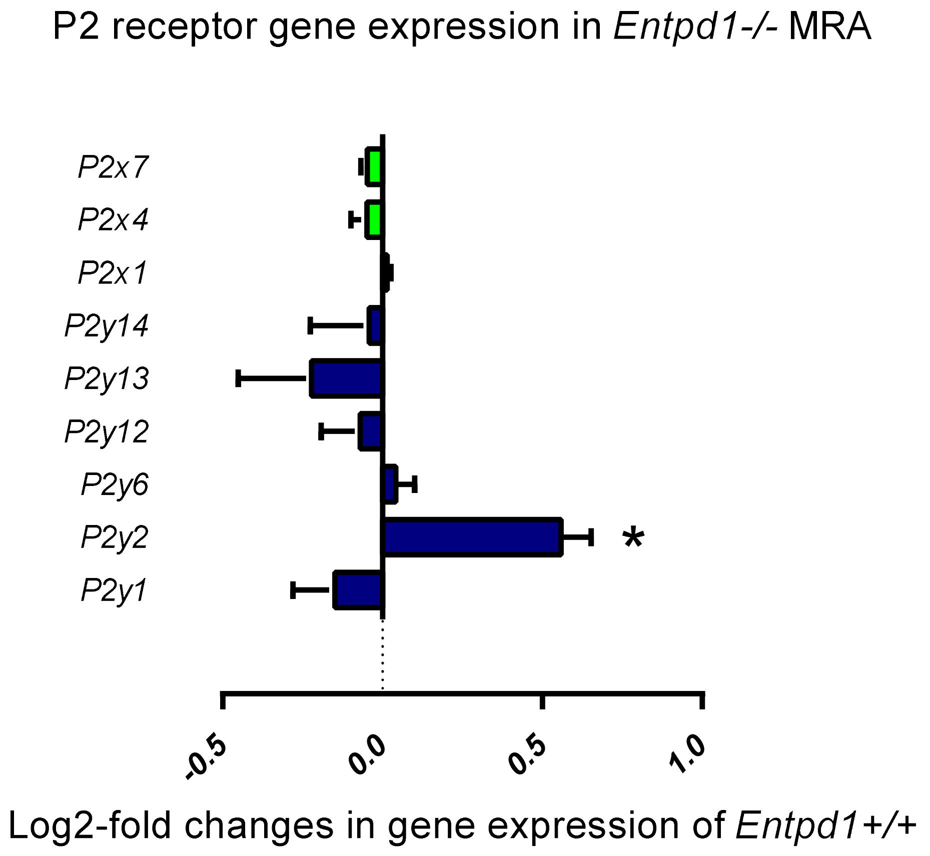

Supplementary Figure S5: P2 receptor gene expression changes in Entpd1-/- MRA compared to WT littermates. Expressions were calculated using the 2ΔCt method (housekeeping genes: Gusb, Hprt, Gapdh). * $p<0.05$ Mann-Whitney test *vs Entpd1+/+* (n=3-5).

Supplementary Figure S6

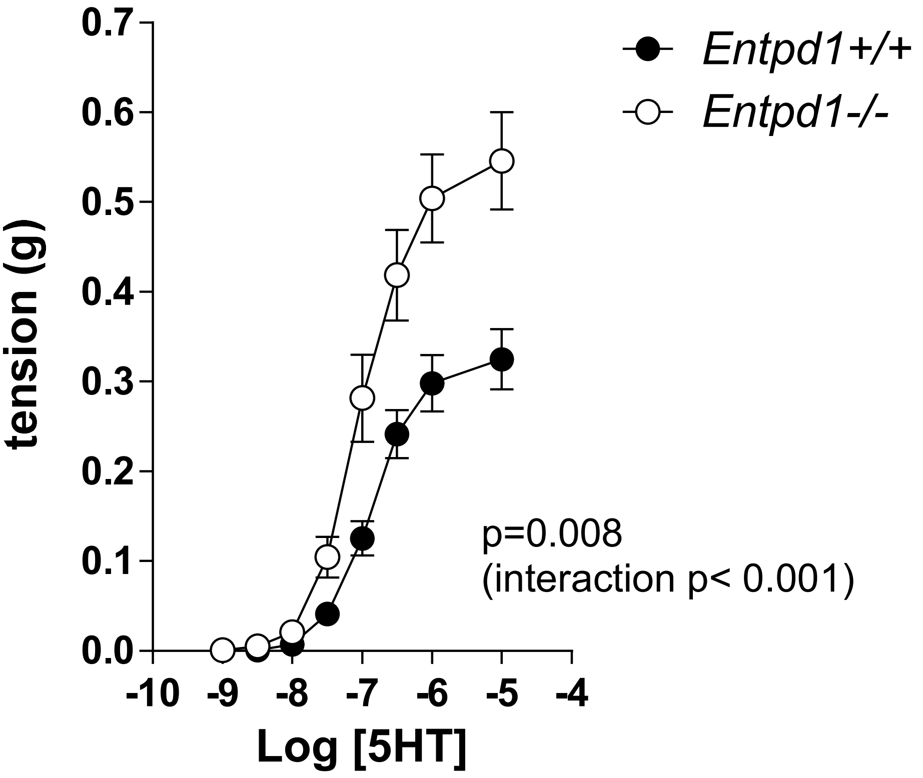

Supplementary Figure S6: *Entpd1-/-* thoracic aortas show greater contraction in response to increasing concentrations of serotonin (5HT) compared to WT controls. Vascular reactivity of isolated aortas was assessed using a wire myograph.. Means ±SEM are shown, n=8-11. Two-way ANOVA for repeated measurements *Entpd1+/+* vs *Entpd1-/-,* p values are shown on graph.

Supplementary Figure S7

A

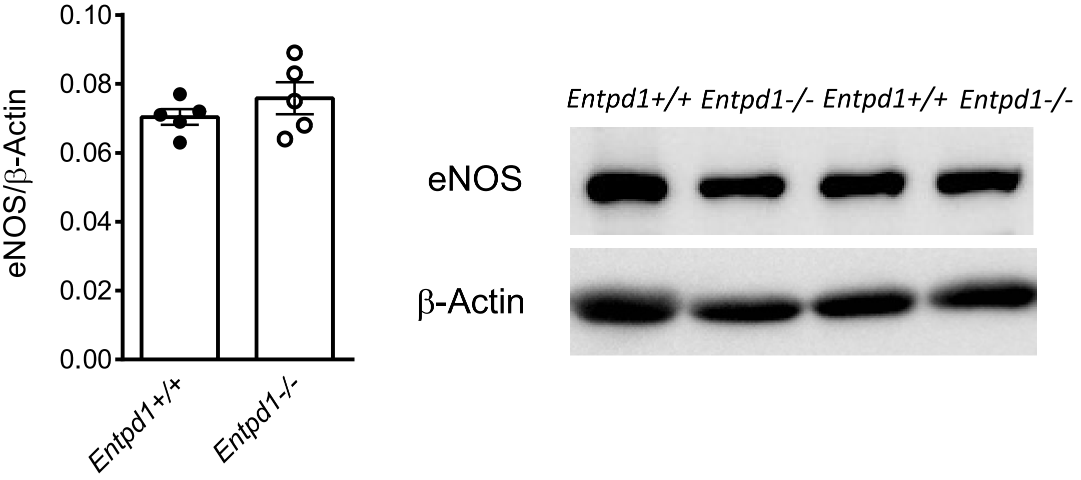

Supplementary Figure S7: Aortic protein eNOS expression. (A) Densitometric quantification (normalized to β-Actin) and representative western-blot analysis of eNOS protein expression (n=5; KO *Entpd1-/-* vs WT *Entpd1+/+* mice). Student t-test $p=0.325$

Supplementary Figure S7

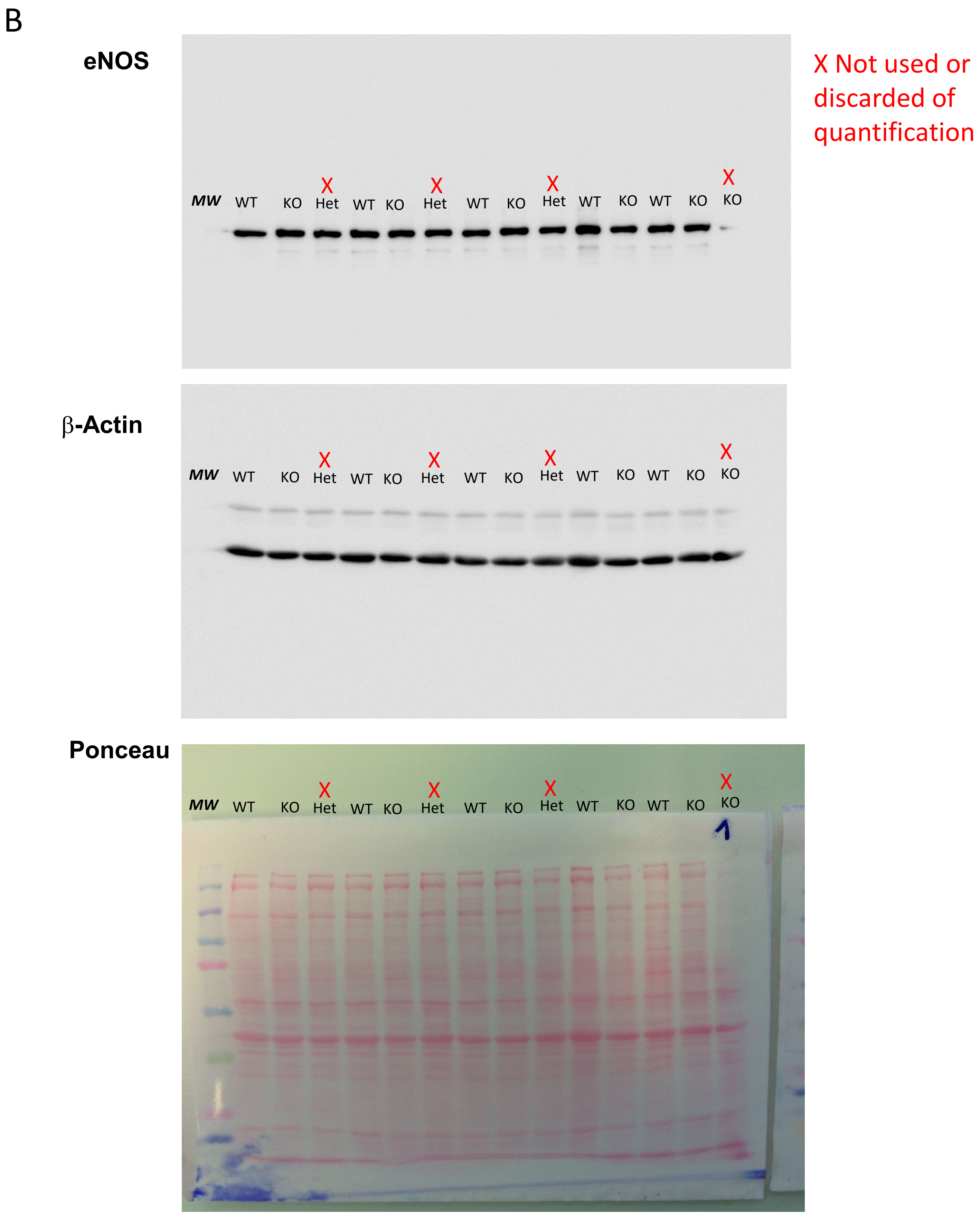

Supplementary Figure S7: (B) Uncropped gels used for western-blot analysis of aortic protein expression of eNOS (140 kD) normalized to β-Actin (42 kD) labeled with primary antibodies anti-eNOS (endothelial nitric oxide synthase; BD Biosciences, Franklin Lakes, NJ; #610297) and anti–β-actin (Sigma, St. Louis, MO; A5316). Control of protein loading and transfer is visualized with ponceau red staining of the nitrocellulose membrane. MW: molecular weight.

Supplementary Figure S8

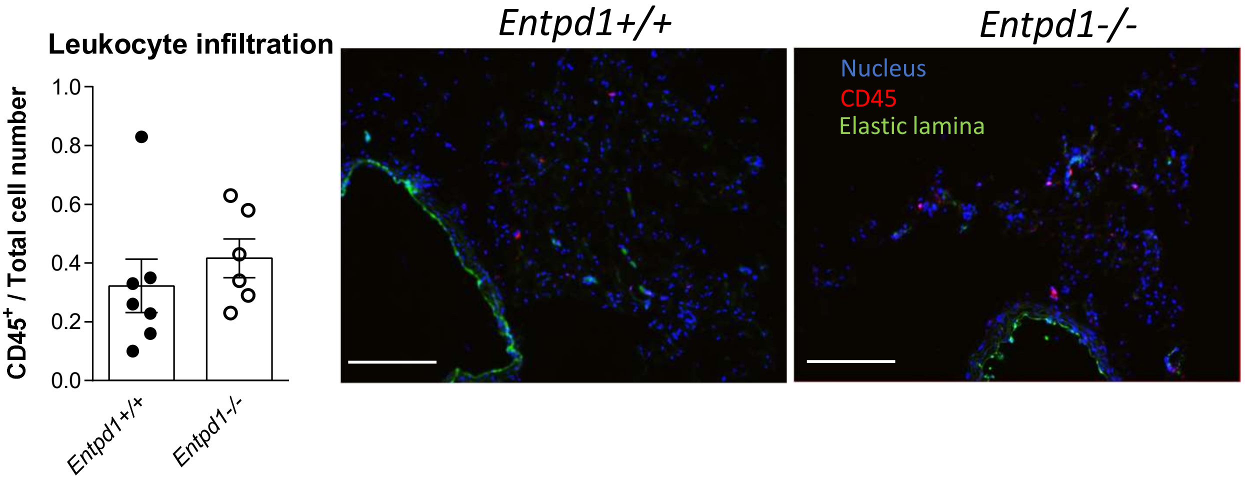

Supplementary Figure S8: Leukocyte infiltration in perivascular area of thoracic aorta. CD45+ cells stained in red were counted and expressed as a ratio of total cell number stained with Dapi in blue. The green autofluorescence represents multilayered lamina. Scale bar 100µm.

		Entpd1+/+		*Entpd1-/-*		
Protein	Gene	Mean	SEM	Mean	SEM	*p*
Inflammation						
CD45	*Ptprc*	2,54E-03	1,85E-04	3,51E-03	4,56E-04	*0,078*
F4/80	*Emr1*	8,72E-03	1,17E-03	8,44E-03	1,08E-03	*0,861*
Cxcr3	*Cxcr3*	6,72E-03	2,09E-03	1,79E-02	4,69E-03	*0,074*
Il-6	*Il-6*	5,07E-03	8,88E-04	1,12E-02	5,22E-03	*0,270*
Mcp1	*Ccl2*	3,97E-02	1,69E-02	1,86E-02	3,66E-03	*0,253*
Cxcl2	*Cxcl2*	2,09E-02	7,51E-03	1,23E-02	2,12E-03	*0,297*
Cox2	*Ptgs2*	1,05E-02	3,58E-03	2,47E-02	4,99E-03	*0,054*
Inducible nitric oxide synthase	*Nos2*	3,75E-03	8,52E-04	3,94E-03	8,19E-04	*0,875*
Oxidative stress						
Gp91-phox	*Cybb*	1,19E-02	2,97E-03	1,42E-02	1,96E-03	*0,524*
Superoxide dismutase-1	*Sod1*	1,02E+01	1,00E+00	1,02E+01	9,48E-01	*1,000*
Superoxide dismutase-2	*Sod2*	9,66E+00	1,07E+00	9,08E+00	1,45E+00	*0,752*
NADPH oxidase 4	*Nox4*	4,69E-02	6,13E-03	5,08E-02	9,02E-03	*0,730*
Cell adhesion						
Vascular cell adhesion molecule-1	*Vcam1*	3,38E-02	5,96E-03	3,29E-02	2,99E-03	*0,901*
Intercellular adhesion molecule-1	*Icam1*	1,45E-02	3,04E-03	1,89E-02	3,86E-03	*0,395*
E-selectin	*Sele*	7,27E-03	1,84E-03	1,30E-02	3,44E-03	*0,172*
Matrix proteolysis						
Matrix metalloproteinase-2	*Mmp2*	3,61E-02	8,44E-03	3,34E-02	4,73E-03	*0,786*
Matrix metalloproteinase-9	*Mmp9*	1,53E-03	2,78E-04	1,13E-03	1,54E-04	*0,265*
Tissue inhibitor of metalloproteinase-1	*Timp1*	2,88E-02	9,14E-03	2,84E-02	3,48E-03	*0,969*
Tissue inhibitor of metalloproteinase-2	*Timp2*	6,77E-01	1,36E-01	6,65E-01	1,12E-01	*0,945*
	Mmp9/Timp1	8,28E-02	3,14E-02	3,65E-02	3,68E-03	*0,217*
	Mmp2/Timp2	5,40E-02	5,55E-03	5,16E-02	4,78E-03	*0,744*
Plasminogen activator Inhibitor-1	*Serpine1*	2,73E-02	8,25E-03	3,17E-02	1,46E-02	*0,799*
Neutrophil elastase	*Elan*	9,05E-03	4,48E-03	7,35E-03	2,79E-03	*0,746*
Cathepsin G	*Ctsg*	1,72E-02	3,87E-03	6,60E-03	2,40E-03	*0,055*
Thromboregulation						
Thrombomodulin	*Thbd*	2,60E-02	5,27E-03	2,79E-02	4,79E-03	*0,793*
Heparan sulfate proteoglycan	*Hspg2*	6,16E-02	1,16E-02	7,51E-02	1,66E-02	*0,521*
Fibrosis						
Connective tissue growth factor	*Ctgf*	6,09E-01	8,88E-02	4,56E-01	5,26E-02	*0,168*
Tissue growth factor-beta-1	*Tgfb*	5,71E-02	1,10E-02	6,33E-02	7,57E-03	*0,653*
Collagen-1	*Coll1A1*	8,81E-02	1,44E-02	8,42E-02	1,76E-02	*0,866*
Vascular homeostasis						
Endothelial nitric oxide synthase	*Nos3*	5,07E-02	9,51E-03	5,35E-02	8,19E-03	*0,827*
Endothelin-1	*Edn1*	3,19E-02	9,97E-03	2,22E-02	3,08E-03	*0,377*
Vascular endothelial growth factor receptor-2	*Kdr*	1,76E-02	2,45E-03	1,91E-02	2,84E-03	*0,688*
Vascular endothelial growth factor-α	*Vegfa*	1,22E-01	2,35E-02	1,28E-01	1,33E-02	*0,815*
Hypoxia inducible factor	*Hif1a*	1,47E-01	1,34E-02	1,67E-01	2,14E-02	*0,458*

Supplementary Table S1: Expression of genes likely to modulate vascular function. mRNA expression was determined in thoracic aorta with perivascular fat of *Entpd1-/-* and *Entpd1+/+* mice (n = 5-6) by RT-qPCR. Data are presented as means ± SEM. *p* represents Student's *t*-test analysis vs. Controls. Methods: Tissues from *Entpd1-/-* and *Entpd1+/+* controls were stored at − 20 °C in RNAlater Stabilization Reagent (Qiagen). RNA extraction was performed with the RNeasy® Micro Kit (Qiagen). RNA extract was used to synthesize cDNA. RT-qPCR was performed with SYBR® Select Master Mix (Applied Biosystems) by using a LightCycler 480 Real-Time PCR System (Roche). mRNA levels were expressed as 2(Ct target gene – Ct mean of reference genes). Reference genes represent the 3 housekeeping genes *Gapdh*, *Gusb* and *Hprt*. Sequences of primer pairs are represented in Supplementary Table S2.

Gene	Protein	Gene ID	Amplicon size	Forward	Reverse
Ccl2 bis	MCP1	NM_011333.3	75	gcctgctgttcacagttgc	caggtgagtggggcgtta
Col1a1	Col1a1	NM_007742.3	94	catgttcagctttgtggacct	gcagctgacttcagggatgt
Ctgf	Ctgf	NM_010217.2	112	tgacctggaggaaaacattaaga	agccctgtatgtcttcacactg
Ctsg	Cathepsin G	NM_007800.2	91	acggttctggaaagatgcag	tctcggcctccaatgatct
Cxcl2 ter	MIP2	NM_009140.2	102	aacatccagagcttgagtgtga	ttcagggtcaaggcaaactt
Cxcr3	Cxcr3 (CD183)	NM_009910	89	gcagcacgagacctgacc	ggcatctagcacttgacgttc
Cybb	Gp91-phox	NM_007807.4	71	gaggttggttcggttttgg	gttttgaaagggtgggtgac
Edn1	Endothelin-1	NM_010104.2	87	tgctgttcgtgactttccaa	gggctctgcactccattct
Ela-2	Neutrophil Elastase	NM_015779.2	94	tggaggtcatttctgtggtg	ctgcactgaccggaaatttag
Emr1	F4/80	NM_010130	113	gcaaggagaatgagtccattaac	tgagacaaaagccactcctg
Entpd1	CD39	NM_009848.3	76	ctcctgcaaggctataacttcac	gcgttgctgtctttgatcttg
Gapdh	GAPDH	NM_008084.2	121	ccggggctggcattgctctc	ggggtgggtggtccagggtt
Gusb	GUSB	NM_010368.1	72	ctctggtggccttacctgat	cagttgttgtcaccttcacctc
Hif1a	HIF1α	NM_010431.2	104	gcactagacaaagttcacctgaga	cgctatccacatcaaagcaa
Hprt	HPRT	NM_013556.2	125	ggggtgggtggtccagggtt	aagacattctttccagttaaagttgag
Hspg2	Hspg2	NM_008305.3	95	caccttcgctggctcaag	tggcagctctggtctaagttc
Icam1	ICAM	NM_010493.2	80	gctaccatcaccgtgtattcg	aggtccttgcctacttgctg
Il6	IL-6	NM_031168.1	84	gatggatgctaccaaactgga	ccaggtagctatggtactccagaa
Kdr	VegfR2	NM_010612.2	65	cagtggtactggcagctagaag	acaagcatacgggcttgttt
Mmp2	MMP2 (gelatinase A)	NM_008610.2	74	gtgggacaagaaccagatcac	gcatcatccacggtttcag
Mmp9	MMP9 (gelatinase B)	NM_013599.2	108	ttctggcacacgcctttc	ccatagtaagtggggatcacg
Nos2	iNOS	NM_010927.3	65	ctttgccacggacgagac	tcattgtactctgagggctgac
Nos3	eNOS	NM_008713.4	65	ccagtgccctgcttcatc	gcagggcaagttaggatcag
Nox4	NOX4	NM_015760.4	69	ctggaagaacccaagttcca	aaaggcacaaaggtccagaa
Nt5e	Cd73	NM_011851.4	76	ccattgatgagaagaacaatggta	gtcaaatgtccctccaaagg
P2rx1	P2X1	NM_008771.3	86	ccgaagccttgctgagaa	ggtttgcagtgccgtacat
P2rx4	P2X4	NM_011026.2	89	ccaacacttctcagcttggat	tggtcatgatgaagagggagt
P2rx7	P2X7	NM_011027.2	76	ctggttttcggcactgga	ccaaagtaggacagggtgga
P2ry1	P2Y1	NM_008772.4	60	ctgtgtggaccccattcttt	tcgggacagtctccttctga
P2ry12	P2Y12	NM_027571.3	94	gagacactcatatccttcagattcag	tcgggacagtctccttctga
P2ry13	P2Y13	NM_028808.3	60	atgtgtgagatggggaaagg	gtcccaggggagaaggtg
P2ry14	P2Y14	NM_001008497.2	59	ctttgcctccagaggtgaga	ggctggtttggagggtct
P2ry2	P2Y2	NM_008773.3	127	tgcgctgatgaactttgttt	ggcaggaaacaggaagaaca
P2ry6	P2Y6	NM_183168.1	80	tcttccatcttgcatgagaca	ggatggtgccattgtcct
Panx1	PANX1	NM_019482.2	61	agaccaagggagaggacca	gctgctcaggtccaaatctt
Ptgs2 bis	COX2	NM_011198.3	109	gggagtctggaacattgtgaa	gcacattgtaagtaggtggactgt
Ptprc	CD45	NM_011210	111	cgggatgagacagttgatga	gtattctgcgcacttgttcct
Sele	E-selectin	NM_011345.2	67	tcctctggagagtggagtgc	ggtgggtcaaagcttcacat
Serpine1	Serpine1 (PAI1)	NM_008871	80	ggcacctttgaatactcagga	tttcccagagaccagaacca
Sod1	SOD1	NM_011434.1	78	caggacctcattttaatcctcac	tgcccaggtctccaacat
Sod2	SOD2 (MnSOD)	NM_013671.3	68	gacccattgcaaggaacaa	gtagtaagcgtgctcccacac
Tgfb1	Tgf-beta1	NM_011577.1	70	tggagcaacatgtggaactc	cagcagccggttaccaag
Thbd	Thrombomodulin	NM_009378.3	62	tggcgataaccacaccagt	agagtggagccgtctggtc
Timp1	TIMP1	NM_011593.2	93	catggaaagcctctgtggat	gatgtgcaaatttccgttcc
Timp2	TIMP2	NM_011594	60	ttttgcaatgcagacgtagtg	ggaatccacctccttctcg
Vcam1	VCAM1	NM_011693.2	110	tgattgggagagacaaagca	aacaaccgaatccccaactt
Vegfa	Vegfa	NM_001025250.3	74	aaacgaaagcgcaagaaatc	atgctttctccgctctgaac

Supplementary Table S2: Sequences of primer pairs used for RT-qPCR.
Housekeeping genes: *Gapdh, Gusb, Hprt.*
